# The status of health promotion lifestyle and its related factors in Shandong Province, China

**DOI:** 10.1186/s12889-021-11152-6

**Published:** 2021-06-15

**Authors:** Qianqian Liu, Shusheng Huang, Xiaoyuan Qu, Aitian Yin

**Affiliations:** 1 Centre for Health Management and Policy Research, School of Public Health, Cheeloo College of Medicine, Shandong University, Jinan, 250012 China; 2 NHC Key Lab of Health Economics and Policy Research (Shandong University), Jinan, 250012 China; 3 Student Counseling Center, Shandong University, Jinan, 250100 China; 4 School of Nursing and Health, Henan University, Kaifeng, 475000 China

**Keywords:** Health attitude, Health promotion lifestyle, Health promotion, Cross-sectional study

## Abstract

**Objectives:**

This study aims to explore the status of Shandong Province, China residents’ health promotion lifestyle and its influencing factors, especially to explore how health attitude affects health promotion lifestyle, thus can make targeted recommendations for health promotion in China and similar areas.

**Methods:**

1800 adults were selected from urban and rural areas of Shandong Province, China, using multistage stratified, cluster random sampling method. A survey was conducted face-to-face from March to May, 2018, using Health Promotion Lifestyle Profile and Health Attitude Questionnaire. The between-group measured data were compared by One-way ANOVA or *t*-tests. The correlation between the health attitude and health promotion lifestyle was examined by Pearson correlation. Logistic regression model was used to examine the related factors influencing health promotion lifestyle. Health promotion lifestyle is the dependent variable, and gender, education level, annual family per capita income and health attitude are the independent variables.

**Results:**

The mean (SD) of HPLP-IICR total score of the participants was 82.12(16.63). 54.50% of the participants had poor or average health promotion lifestyle, while 45.50% had good or excellent health promotion lifestyle. Significant differences existed in health promotion lifestyle among different gender, education level, income level, marital status, and health attitude (*Ps* < 0.001). Multivariable Logistic regression model found that male (OR = 0.35, 95% CI: 0.12–0.34), high school education level (OR = 0.57, 95% CI:0.17–0.41), junior middle school & below (OR = 0.42; 95% CI:0.12–0.33), annual family per capita income with < 10,000 CNY (OR = 2.53, 95% CI:1.24–2.06; OR = 2.14, 95% CI:1.08–3.12), low health affection (OR = 0.39, 95% CI:2.15–4.22), and low health behavioral intention (OR = 0.21; 95% CI: 2.33–5.29) were statistically significant correlates of average or poor health promotion lifestyle.

**Conclusions:**

The health lifestyle needs to be further promoted in Shandong Province, China. The government and social sectors are encouraged to make more efforts to improve the accessibility and quality of health services. Meanwhile, individual responsibility cannot be ignored as well. More affective factors and operable measures should be added to enhance health affection and health behavioral intention, so as to enhance health promotion lifestyle.

## Introduction

Several studies have demonstrated that lifestyle plays a vital role in explaining health and disease, especially concerning the onset of disease, seeking help, disease management, and health outcomes [1–3]. Recent research revealed that the two biggest killers of human health are cigarette smoking and physical inactivity, accounting for more than 800,000 premature deaths in the United States annually and exerting enormous adverse economic impact [4]. Poor lifestyle is the pathogenic factor constituting 70% of the top ten causes of diseases in the United States, while in China, the corresponding figure is 44.7% [5]. In China, three decades of unprecedented, rapid economic development have resulted in high rates of urbanization and accelerated advances in technology, which in turn have led to major changes in the health lifestyle [6, 7] of more than 1.3 billion people [8]. The resulting social-economic transformation has caused Chinese society to gradually adopt an unhealthy lifestyle [9]. That can increase the possibility of developing unhealthy body weight [10, 11], which can in turn lead to an increased health risk for hyperlipidemia, high blood pressure, type 2 diabetes, low bone density, and other diseases [12]. Previous study demonstrated that among older Chinese adults (age ≥ 60 years), the overall prevalence of multimorbidity was up to 87.0% in urban residents [13], while the proportion was even up to 90% [14] in rural residents. The medical burden of chronic diseases has become the largest proportion of medical expenses of rural residents in China [15].

Previous studies on Chinese health promotion lifestyle mainly focused on the middle-aged, the elderly [6, 11, 16–19] or the youth [9, 20], while few studies covered all adults. Most of them focused on the social demographic influential characteristics, such as gender, age, residence, marital status and education level [11, 16], which are important for developing health promotion interventions. Besides, health promoting lifestyle is also influenced by individual psychological factors, such as ‘attitude’ [21], one of the core theoretical constructs in the Theory of Planned Behavior [22] and other health behavior theories [23]. A majority of research suggests a significant link between attitude and health promotion lifestyle [24–26]. Though an influential theory suggests that the structure of attitude includes three components—cognition, affection, and behavioral intention [27–29], many previous studies only focused on the cognitive aspect [25, 26, 30], overlooking the affection and behavioral intention which cannot present the whole attitude. Some research based on KAP theory even equated attitude with knowledge [25, 26, 30].

The hypothesis of this study is that the social demographic characteristics and three components of health attitude affect the health promotion lifestyle. By examining the health promotion lifestyle of broader Chinese population, more specific social demographic influential characteristics can be found. Meanwhile, exploring the influence of health attitude on health promotion lifestyle from three aspects can suggest new integrative and targeted ways for promoting health lifestyle. Relevant suggestions will be proposed to residents of China and similar areas to form health promotion lifestyle.

## Methods

### Study design

The study was conducted on the residents of Shandong Province in the eastern region of China. Shandong is an economically developed province of China, ranking third in China’s GDP in 2018 [31]. By the end of 2017, the permanent resident population of Shandong Province was 10.0583 million [32]. Shandong Province has put great emphasis on residents’ medical concern for several years, and has established a basic medical care system [33]. Furthermore, the economic status and employment category of residents can represent the status of the majority of Chinese residents. The survey was conducted in community health centers or township health centers from March to May, 2018. Before the survey, the investigators had been trained to conduct preliminary publicity and introduction. The survey was conducted anonymously and the participants were patiently told that filling in the questionnaire would not have any impact on them. After the survey, household cleaning products worth 20CNY were provided to the participants as a reward.

### Participant

The study sample was selected using multistage stratified, cluster random sampling method. Based on the level of socioeconomic development, Jiaonan, Qufu, and Ningyang of Shandong Province were selected as sample counties. Based on the level of economic development, one street, one town, and three villages in each city/county were selected randomly for research after consulting the local health bureau management, 600 people were selected from each city/county, adding up to 1800 people in total.

### Data collection tools

#### Basic sociodemographic characteristics

The questionnaire mainly involves gender, age, annual family per capita income, marital status, residence, education level, whether suffering from chronic diseases including cardiovascular disease, cancer, chronic respiratory disease and diabetes [34], and medical insurance status.

#### Health promotion lifestyle profile

We used the Chinese version of the Health Promotion Lifestyle Profile (HPLP-IICR) [35]. The original version of the HPLP was developed by Walker et al. [36, 37], contains 52 items. The Chinese version of the HPLP-IICR has been simplified by Yen [35], which contains 30 items, and five subscales, including Spiritual Growth (6 items), Physical Activity (6 items), Health Management (9 items), Nutrition (5 items), and Health Responsibility (4 items). A higher score indicates greater health promotion, using a 4-point Likert scale with choices ranging from never = 1 to always = 4. The overall Cronbach’s alpha is 0.90, with 0.69 to 0.87 for the subscales [35]. In the present study, the expression mode of some items has been adjusted to make it easy to be understood. Adjusted Cronbach’s alpha for the total profile is 0.88, with subscales ranging from 0.71 to 0.85. The total score ranges between 30 and 120, in which 30–52 represents poor, 53–75 represents average, 76–98 represents good, and 99–120 represents excellent.

#### Health attitude questionnaire

A self-designed Health Attitude Questionnaire was used in the study, which using a 5-point Likert scale with choices ranging from totally disagree = 0 to totally agree = 4 [38, 39]. The original questionnaire was designed according to health promotion lifestyle, contained 30 items. The final questionnaire contains 23 items and, the Cronbach’s α is 0.81. We extracted three factors--Health Cognition (6 items), Health Affection (8 items), and Health Behavioral intention (9 items), which respectively explained 15.37, 14.71, and 23.80% of the total variance. The confirmatory factor analysis of the three-factor model resulted in an acceptable model fit (Tucker–Lewis Index = 0.90, Comparative Fit Index = 0.92, Normed Fit Index = 0.93, Expected Cross-Validation Index = 0.35, Root-Mean-Square Error of Approximation = 0.068). The item loadings range from 0.72 to 0.83, and correlation coefficients among the three factors range from 0.54 to 0.70. The total score is between 0 and 92, in which 0–30 represents poor, 31–61 represents average, and 62–92 represents good.

### Ethics

The study was conducted and approved by Ethics Committee of Centre for Health Management and Policy Research, School of Public Health, Cheeloo College of Medicine, Shandong University. All the participants had given their informed consent. The study was conducted according to the guidelines of the Declaration of Helsinki.

### Data analysis

In this study, the database was built using Access 2010 software; the data was recorded two times and compared to ensure data integrity and accuracy. The statistical analysis was performed using the Statistical Package for the Social Sciences version 24.0 (SPSS, Inc., Chicago, IL, USA). The independent variables of this study are individual-level factors including gender (male/female), age (< 30/30–50/> 50), education level (university and above/ high school/junior middle school and below), chronic disease (yes/no), marital status (married/unmarried or divorced/ widowed), and medical insurance (Urban and Rural Basic Medical Insurance/Urban Employee Medical Insurance/uninsured), all of which were summarized using descriptive statistics. Standard 5 points was used to standardize the different scores of the subscale for comparison. Besides, the analysis of variance or *t*-test was used to compare the between-group measured data. All tests were bilateral, and the test level was set at *α* = 0.05. The Pearson correlation coefficient was used to analyze the correlation between the health attitude and health promotion lifestyle. Multivariate logistic regression model was used to explore the related factors affecting Chinese residents’ health promotion lifestyle. With health promotion lifestyle (0 = excellent and good, 1 = average and poor) as the dependent variable, significant factors in single-factor analysis were selected as independent variables for multivariate logistic regression analysis, which include gender, education level, annual family per capita income, marital status, and three factors of health attitude: health cognition, health affection, and health behavioral intention. The results were exhibited as adjusted odds ratios (ORs) with their 95% confidence intervals (CIs).

## Results

### Basic sociodemographic characteristics

In this study, 1800 questionnaires were distributed, of which 1784 were collected, with 1769 valid responses (effective rate, 98.28%). Among the 1769 respondents, 790 (44.66%) are male and 979 (55.34%) are female; the mean age is 45.6 (SD = 9.12). Table 1 presents the sociodemographic data of study participants.

**Table 1:** Characteristics of study participants (*n* = 1769)

Variables	*N* (%)	Variables	*N* (%)
Age		Education level	
< 30	133 (7.5)	University and above	598 (33.8)
30–50	1243 (70.3)	High school	724 (40.9)
> 50	393 (22.2)	Junior middle school and below	447 (25.3)
Annual family per capita income (CNY)		Marital status	
< 10,000	506 (28. 6)	Married	1396 (78.9)
10,000-20,000	587 (33. 2)	Unmarried/Divorced	182 (10.3)
> 20,000	676 (38.2)	Widowed	191 (10.8)
Chronic disease		Medical insurance	
No	957 (54.1)	Urban and Rural Basic Medical Insurance	1244 (70.3)
Yes	812 (45.9)	Urban Employee Medical Insurance	506 (28.6)
		Uninsured	19 (1.1)

### Status of health promotion lifestyle

The mean (SD) of HPLP-IICR total score of participants was 82.12(16.63). In all subjects, 254 scored poor, accounting for 14.36%; 710 scored average, accounting for 40.14%; 612 scored good, accounting for 34.60%; 193 scored excellent, accounting for 10.91%. The total score ranges between 30 and 120, in which 30–52 represents poor, 53–75 represents average, 76–98 represents good, and 99–120 represents excellent [35]. After standardized calculation, the mean (SD) of items was 2.73(0.78), 3.08 (0.74) for Nutrition 2.97 (0.79) for Physical Activity, 2.85 (0.78) for Health Management, 2.60 (0.88) for Spiritual Growth, and 2.42 (0.89) for Health Responsibility. As shown in Table 2, one-way ANOVA and *t*-test were used to compare differences in health promotion lifestyle among residents with different characteristics. The residents’ health promotion lifestyle was significantly different (*Ps* < 0.001) among different genders, education levels, annual family per capita income, marital status, and health attitude. Female were found scored higher than male on all subscales except Nutrition. Residents with education level of university or above were scored higher than others on Spiritual Growth, Physical Activity, Nutrition and total score of scale. Residents with Annual family per capita income > 20,000 RMB were found scored higher than others on all subscales except Spiritual Growth. Widowed were found scored lower than married or unmarried/divorced on all subscales, while married were scored higher than unmarried/divorced on Health Management and Health Responsibility, but lower on Physical Activity. Residents with poor, average and good health attitude were found significant differences in the scores of all subscale except Nutrition, showing a gradually increasing trend.

**Table 2:** One-way ANOVA or t-test of different sociodemographic factors on health promotion lifestyle and attitude in Chinese residents

Variables	Spiritual Growth Mean ± SD	Physical Activity Mean ± SD	Health Management Mean ± SD	Nutrition Mean ± SD	Health Responsibility Mean ± SD	Health Promotion Lifestyle Mean ± SD
Gender						
Male	2.58 ± 0.53	2.86 ± 0.85	2.76 ± 0.98	3.12 ± 0.54	2.28 ± 0.68	78.22 ± 19.56
Female	2.71 ± 0.93	3.04 ± 0.74	2.93 ± 0.93	3.09 ± 0.44	2.47 ± 0.94	86.12 ± 14.32
*P*	< 0.001	< 0.001	< 0.001	0.482	< 0.001	< 0.001
Age						
< 30	2.89 ± 0.87	3.01 ± 0. 61	2.88 ± 0.72	3.04 ± 0.98	2.38 ± 0.88	83.76 ± 15.07
30–55	2.56 ± 0.63	2.57 ± 0.43	2.84 ± 0.75	3.01 ± 0.53	2.47 ± 0.77	82.72 ± 14.41
> 55	2.78 ± 0.56	2.98 ± 0.94	2.79 ± 0.71	3.22 ± 0.81	2.41 ± 0.56	82.89 ± 13.91
*P*	< 0.001	< 0.001	0.341	< 0.001	0.071	0.122
Residence						
Rural	2.41 ± 0.88	2.99 ± 0.97	2.92 ± 0.91	3.04 ± 0.91	2.39 ± 0.75	82.05 ± 14.02
Urban	2.76 ± 0.85	2.89 ± 0.82	2.77 ± 0.98	3.10 ± 0.54	2.45 ± 0.72	83.17 ± 15.18
*P*	< 0.001	0.055	< 0.001	0.071	0.229	0.157
Education level						
Junior middle school & below	2.63 ± 0.98	2.67 ± 0.53	2.58 ± 0.90	2.82 ± 0.74	2.40 ± 0.79	80.92 ± 1 4.78
High school	2.58 ± 0.97	2.87 ± 0.84	3.02 ± 0.83	2.99 ± 0.78	2.41 ± 0.82	82.54 ± 1 3.78
University & above	2.75 ± 0.89	2.97 ± 0.87	2.79 ± 0.93	3.12 ± 0.85	2.46 ± 0.85	84.16 ± 16.85
*P*	< 0.001	< 0.001	< 0.001	< 0.001	0.331	< 0.001
Annual family per capita income						
< 10,000 RMB	2.56 ± 0.97	2.94 ± 0.88	2.76 ± 0.83	2.91 ± 2.01	2.14 ± 2.78	80.95 ± 14.78
10,000–20,000 RMB	2. 60 ± 0.92	2.93 ± .0.77	2.84 ± 0.65	3.02 ± 1.78	2.39 ± 1.83	80.76 ± 1 3.94
> 20,000 RMB	2.64 ± 0.91	3.13 ± 0.93	2.95 ± 0.77	3.16 ± 2.22	3.52 ± 2.18	87.01 ± 14.91
*P*	0.663	< 0.001	< 0.001	< 0.001	< 0.001	< 0.001
Marital status						
Married	2.68 ± 0.82	2.66 ± 0.79	3.07 ± 0.99	3.06 ± 0.74	2.51 ± 0.91	85.29 ± 15.01
Unmarried/Divorced	2.60 ± 0.98	3.11 ± 0.93	2.88 ± 0.75	3.09 ± 0.80	2.47 ± 0.79	83.18 ± 13.84
Widowed	2.46 ± 0.90	2.58 ± 0.84	2.62 ± 0.96	2.91 ± 0.77	2.36 ± 0.64	80.96 ± 13.10
*P*	< 0.001	< 0.001	< 0.001	0.056	< 0.001	< 0.001
Chronic diseases						
No	2.72 ± 0.86	2.95 ± 0.68	2.56 ± 0.80	3.06 ± 0.73	2.36 ± 2.96	81.90 ± 1 5.16
Yes	2.55 ± 0.75	2.98 ± 0.92	2.98 ± 0.78	3.11 ± 0.85	2.58 ± 2.29	82.89 ± 1 4.03
*P*	< 0.001	0.696	< 0.001	0.779	< 0.001	0.364
Health attitude						
Poor	2.52 ± 0.61	2.92 ± 0.98	2.75 ± 0.72	3.03 ± 0.66	2.16 ± 0.44	80.98 ± 15.25
Average	2..68 ± 0.79	2.98 ± 0.88	2.78 ± 0.82	3.06 ± 0.88	2.44 ± 0.79	81.77 ± 16.77
Good	2.82 ± 0.75	3.05 ± 0.71	2.98 ± 0.88	3.10 ± 0.75	2.62 ± 0.77	86.44 ± 1 4.13
*P*	< 0.001	< 0.001	< 0.001	0.083	< 0.001	< 0.001

### Correlation analysis of health attitude and health promotion lifestyle

As shown in Table 3, the three aspects of health attitude are correlated with multiple aspects of health promotion lifestyle, suggesting that health attitude is a significant correlate of health promotion lifestyle. Among them, health promotion lifestyle has the highest correlation with health affection and the lowest correlation with cognition.

**Table 3:** Correlation analysis of health attitude and health promotion lifestyle

Variables	Spiritual growth	Physical activity	Health management	Nutrition	Health responsibility	Health promotion lifestyle
Cognition	0.03	0.06*	0.07*	0.20**	0.02	0.06*
Affection	0.08	0.37**	0.33*	0.10*	0.30*	0.38**
Behavioral intention	0.34**	0.44**	0.09*	0.41**	0.12**	0.33**
Health attitude	0.28	0.38**	0.10*	0.36**	0.34*	0.32**

**P < 0.01, *P < 0.05

### Logistic regression analysis of relevant factors influencing health promotion lifestyle

Results indicated that the independent factors influencing health promotion lifestyle include gender, education level, annual family per capita income, health affection, and health behavioral intention. As shown in Table 4, The findings suggested that male (OR = 0.35, 95% CI: 0.12–0.34), high school education level (OR = 0.57, 95% CI:0.17–0.41), junior middle school & below (OR = 0.42; 95% CI:0.12–0.33), annual family per capita income with < 10,000 CNY (OR = 2.53, 95% CI:1.24–2.06; OR = 2.14, 95% CI:1.08–3.12), low health affection (OR = 0.39, 95% CI:2.15–4.22), and low health behavioral intention (OR = 0.21; 95% CI: 2.33–5.29) were statistically significant correlates of average or poor health promotion lifestyle. Female, university & above, annual family per capita income>1000CNY, high health affection, and high health behavioral intention were statistically significant correlates of excellent or good health promotion lifestyle.

**Table 4:** Logistic regression analysis of relevant factors affecting health promotion lifestyle in Chinese residents

Variables	Reference group	B	SE	Wald	*P*	OR	95% CI	
							Lower limit	Upper limit
Gender								
Male	Female	−1.397	0.295	36.61	< 0.001	0.35	0.12	0.34
Education level								
High school	University & above	−1.471	0.278	15.18	0.002	0.57	0.17	0.41
Junior middle school & below		−1.636	0.293	19.21	< 0.001	0.42	0.12	0.33
Annual family per capita income								
> 20,000 CNY	< 10,000 CNY	1.324	0.264	10.25	0.002	2.53	1.24	2.06
10,000–20,000 CNY		0.928	0.377	11.83	< 0.001	2.14	1.08	3.12
Health cognition								
Low grouping	High grouping	−0.687	0.473	11.06	0.055	0.08	1.68	3.22
Health affection								
Low grouping	High grouping	−1.208	0.241	12.24	0.001	0.39	2.15	4.22
Health behavioral intention								
Low grouping	High grouping	−1.325	0.268	13.15	< 0.001	0.21	2.33	5.29

## Discussion

The study focused on assessing health promotion lifestyle of adults in Shandong Province, China, and how health attitude affects health promotion lifestyle. The general situation of their health promoting lifestyle is not excellent. That is consistent with previous studies of China [16–18, 40]. Unlike previous studies, which focused on the middle-aged, the elderly [16–18], and the people who participated in the physical examination [40], this study covered a wider population to represent the overall characteristics of Chinese adults.

At a relative level, the score of Nutrition is the highest, which is the same as the research results of Chen and Zhao [17, 19]. With the increasing improvement of living standards and the widespread nutrition knowledge, Chinese residents have higher requirements on diet, not only to satisfy their appetite, but also to pay more attention to nutrition ingredients and dietary collocation [19].

Different from the relatively low level of Physical Activity in previous studies [18, 40], the Physical Activity level in this study was the second highest. The reason may be due to the differences between the scales used in this survey and those used in other surveys. After revision, the items that do not conform to Chinese culture, such as heart rate self-monitoring, achieving target heart rates, had been deleted, so that they were easier to be understood [35]. It was worth noting that Chinese women showed better Physical Activity than men, which was different from studies of South Koreans [41] and Americans [42]. Perhaps this is because several Chinese women went square dancing as a way of exercise. Square dancing refers to dancing in an open and public space in China, usually on a flat patch such as a public square [43]. This group exercise has become popular in both urban and rural regions across China in recent 15 years [44, 45] and has been popular, mainly among women [43]. Thanks to its convenience, low cost and social function, square dancing has enabled Chinese women to get physical activity and spiritual satisfaction after work and housework [46]. Consistent with many previous studies [47–49], the results of the data analyses show that unmarried or divorced individuals are more likely to engage in physical activity than their married counterparts. In addition to work, married people take more family-oriented economic responsibilities and the responsibility of raising children. For both men and women it is assumed that experiencing multiple simultaneous events has an adverse effect on physical activity participation [49]. This result is also consistent with the model established by Humphreys and Ruseski [50], which found married people spend considerably less time engaged in physical activity each week than unmarried people do. This model also indicates that the time spent on physical activity has a positive relation with education level. This is also consistent with the result that health promotion behavior has a positive relation with education level.

The score of Health Responsibility got the lowest level, similar results had been reported by Zhang [51], whose study is also based on residents of northern China. The protection of health and the self-management of chronic disease can have a positive relation with personal health responsibility [52]. Furthermore, the notion of individual responsibility is believed to be necessary for promoting the justice of a healthcare system [53]. Therefore, this result argues that individual responsibility should be introduced when designing health promotion program. And it should be equally highlighted as the duties of government [53].

A significant work of this study is to discover how health attitude influences health promotion lifestyle. Our main finding is that three aspects of health attitude have certain influence on health promotion lifestyle, among which health cognition has the least influence, followed by health affection, and health behavioral intention has the greatest influence. The influence of health cognition on health promotion lifestyle has been discussed in many previous studies [54]. As the results of these studies showed that, knowledge alone is not sufficient to motivate a change in behavior [55, 56]. This study has found that health affection plays a more important role in the initiation of health promotion lifestyle than health cognition, that was consistent with studies of Lawton [57] and Phipps [58]. In the study of Lawton, a series of hierarchical regression analyses revealed that affective attitude was a significantly more powerful predictor of behavior than cognitive attitude for 9 behaviors [57]. Phipps found that physical activity was predicted by affective but not instrumental attitude, and through the implicit and explicit measures, implicit affective attitude was found to significantly predict physical activity behavior independently of explicit affective attitude and consciously held intention to engage in physical activity [58] Some of previous studies suggested that intention is also an established correlate of health behavior, yet discordance is considerable in experimental research. Some meta-analysis demonstrated a weak relationship between intention and behavior that may be below meaningful/practical value [59, 60]. The results of this study support the influence of health intention on health promoting lifestyle.

In addition to attitude, this study found that female residents may adopt more health promotion lifestyle, corroborating previous studies [61, 62]. The effects of education and family income on health promotion lifestyle are similar to previous studies [63, 64]. Prior studies suggested that poorly educated adults may have difficulty in understanding medical statistics, drug dose requirements, and basic health concepts such as daily nutritional value [65–67]. People with a high school diploma or above, tend to seek and use health information, regardless of their educational level and other sociodemographic factors [68, 69]. Bourdieu’s study [70] also indicated that the higher the socio-economic status, the healthier the lifestyle.

In China, as in other countries, inconsistencies in health behavior due to differences in gender, wealth, and education [71] still persist; thus, decreasing the gap should be a top issue of particular urgency to both public and private sectors [72]. Meanwhile, individual health responsibility should be emphasized [73]. Due to the important influence of health attitude on health promotion lifestyle, affection and behavioral intention should be fully considered in the process of health promotion. For example, given the increasing use of social media by Chinese residents [74], its use may be considered a judicious way to promote health lifestyle or health information [75], it can improve the accessibility of health information to various population groups—regardless of age, education, income, and locality to health information—compared to traditional communication methods [76–78]. It can also provide valuable social and affective support to users [75, 79], it is necessary to design participatory health promotion measures based on social media. Based on influence of health intention on health promotion lifestyle, practical activities or health-relevant motor manipulations should be designed in facilitating health lifestyle [80]. In addition, in order to make up for the lack of physical activity among married people, health promotion projects requiring family participation could be designed.

### Limitations

The findings of this study should be considered in light of some limitations. Health attitude and health promotion lifestyle are self-reported variables, so that social desirability bias may exist. In the study design, other psychological features of individuals were not involved except health attitude, which limited the analysis of health promotion lifestyle. However, despite these limitations, this study can reflect the current health promotion lifestyle of residents of Shandong Province, China and, reveal the influence of health attitude on health promotion lifestyle. It also provides relevant suggestions for the formulation of relevant health policies.

## Conclusions

This study found that the general health promotion lifestyle in Chinese residents is not excellent. Of note, gender, education level, annual family per capita income, health affection, and health behavioral intention are significant factors influencing residents’ health promotion lifestyle. Furthermore, this study suggests that more affective factors and operable measures should be added to enhance health affection and health behavioral intention. The government, medical institutions and individuals should take their own responsibilities to form health promotion lifestyle.

## Data Availability

The datasets used during the current study are available from the corresponding authors on reasonable request.

## Abbreviations

WHO: World Health Organization
HPLP: Health Promotion Lifestyle Profile
